# Effect of ultrasonic degradation on the physicochemical property and bioactivity of polysaccharide produced by *Chaetomium globosum* CGMCC 6882

**DOI:** 10.3389/fnut.2022.941524

**Published:** 2022-07-19

**Authors:** Shiwei Li, Yingna Wang, Weipeng Dun, Wanqing Han, Chunping Xu, Qi Sun, Zichao Wang

**Affiliations:** ^1^College of Life Sciences and Agronomy, Zhoukou Normal University, Zhoukou, China; ^2^College of Food Science and Technology, Henan University of Technology, Zhengzhou, China; ^3^College of Food and Bioengineering, Zhengzhou University of Light Industry, Zhengzhou, China; ^4^College of Life Sciences, Chongqing Normal University, Chongqing, China; ^5^School of Biological Engineering, Henan University of Technology, Zhengzhou, China

**Keywords:** *Chaetomium globosum* CGMCC 6882, polysaccharide, ultrasonic degradation, molecular weight, biological activity

## Abstract

Similar to the enzymatic process, there might also be an active fragment in polysaccharides, how to obtain is important for investigating the bioactivity and pharmacological mechanism of polysaccharides. Presently, a *Gynostemma pentaphyllum* endophytic fungus *Chaetomium globosum* CGMCC 6882 polysaccharide [Genistein Combined Polysaccharide (GCP)] was degraded by ultrasonic treatment, two polysaccharide fragments of GCP-F1 and GCP-F2 were obtained. Physicochemical results showed that GCP-F1 and GCP-F2 had the same monosaccharide composition of arabinose, galactose, glucose, xylose, mannose, and glucuronic acid as compared to GCP with slightly different molar ratios. However, weight-average molecular weights of GCP-F1 and GCP-F2 decreased from 8.093 **×** 10^4^ Da (GCP) to 3.158 × 10^4^ Da and 1.027 × 10^4^ Da, respectively. *In vitro* scavenging assays illustrated that GCP-F1 and GCP-F2 had higher antioxidant activity against 2,2′-azinobis-(3-ethylbenzothiazoline-6-sulfonic acid (ABTS) radical, 2,2-diphenyl-1-picrylhydrazyl (DPPH) radical, superoxide anions, and hydroxyl radical than GCP, the order was GCP < GCP-F1 < GCP-F2. Meanwhile, antibacterial tests showed that ultrasonic degradation increased the antibacterial activity of GCP-F1 as compared to GCP, but GCP-F2 almost lost its antibacterial activity with further ultrasound treatment. Changes in the antioxidant and antibacterial activities of GCP-F1 and GCP-F2 might be related to the variation of their molecular weights.

## Introduction

As a macromolecule consisting of more than ten monosaccharides through α-glycosidic or β-glycosidic bonds, polysaccharide is one of the essential substances for organism and has many bioactivities, such as antioxidant, antibacteria, anti-inflammatory, antidiabetic, antitumor, regulating intestinal micro-ecology, improving immunity, and promoting growth (1, 2). Even in fighting against COVID-19, polysaccharide also shows an excellent potential application (3). Meanwhile, polysaccharide also has good applications in food, breeding, planting, industrial, and agricultural fields. For instance, *Mesona chinensis* Benth polysaccharide could improve the pasting viscosity, viscoelasticity, gelling properties, and water-retention capacity of rice starch and shows a good prospect of application in food modification (4). Chitosan oligosaccharides could destroy cellular permeability and alter cellular metabolism of food spoilage fungi *Aspergillus Flavus* and *Aspergillus Fumigatus* and potentially be used as antiseptics in food (5). Xantho-oligosaccharide could inhibit phytopathogenic *Xanthomonas campestris* pv. *campestris* and be used as a biopesticide in the planting industry (6). *Ganoderma lucidum* polysaccharide could promote pig health and improve pork quality and be used as a potential feed additive in the breeding industry (7).

The structural feature of polysaccharide is the basis of its biological activity, and the structural difference will endow polysaccharide with different bioactivities. For example, the fucose ratio in the monosaccharide composition of *Pleurotus geesteranus* polysaccharide affected its antioxidant and hepatoprotective effects (8). Triple-helix lentinan exhibited the highest antitumor activity against *Sarcoma* 180 solid tumor *in vivo* (9). Spherical molecular shape was important for apoptosis-inducing activity of *Lycium barbarum* polysaccharide against human hepatoma cells (10). The sulfated group and β-glycosidic bond affected the antitumor activity of exo-polysaccharides from *Lactobacillus plantarum* 70810 (11). Meanwhile, molecular weight influenced the hypoglycemic effects of konjac glucomannan on type 2 diabetic rats (12). Homoplastically, almost all structural characteristics of polysaccharides affect their biological activities, in addition to the detection and analysis methods of polysaccharides lagging behind protein and DNA, it is difficult to uncover the relationship between structural features and biological activities of polysaccharides presently. Based on this, we select the easily controlled structural feature of molecular weight and study its effect on polysaccharide bioactivity (13), thus providing guidance for the analysis of the structure-activity relationship of polysaccharides.

*Chaetomium globosum* CGMCC 6882 was an endophytic fungus extracted from *Gynostemma pentaphyllum*, which could use some carbon sources to produce different bioactive polysaccharides (14, 15), but how structure affects the biological activity of polysaccharides produced by *C. globosum* CGMCC 6882 was not clear. Meanwhile, Zhang et al. (16) found that molecular weight influenced the activity of polysaccharides produced by *C. globosum* CGMCC 6882. Therefore, an antibacterial polysaccharide of Genistein Combined Polysaccharide (GCP) produced by *C. globosum* CGMCC 6882 was degraded by ultrasonic treatment in the present work for seeking the potential active fragment. On the one hand, physicochemical properties of GCP before and after ultrasonic degradation were compared. On the other hand, activities of pristine and ultrasound-degraded GCP fragments were assessed.

## Materials and methods

### Materials and chemicals

Production, extraction, and purification of polysaccharide GCP produced by *C. globosum* CGMCC 6882 were conducted according to the methods previously reported (1). Standard monosaccharides of fucose, rhamnose, arabinose, glucosamine, galactose, glucose, xylose, mannose, fructose, galacturonic acid, and glucuronic acid were bought from Sigma-Aldrich (Sigma Chemicals, St. Louis, United States). Dextran standards were purchased from Shanghai Aladdin Biochemical Technology Co., Ltd. (Shanghai, China). Trifluoroacetic acid (TFA), potassium bromide (KBr), 2,2-diphenyl-1-picrylhydrazyl (DPPH), ascorbic acid (Vc), and other chemicals were purchased from Sinopharm Chemical Reagent Co. (Beijing, China).

### Ultrasonic degradation of Genistein Combined Polysaccharide

Ultrasound degradation of GCP was carried out on a JY98-IIIDN Ultrasonic Processor (Xinzhi Bio-Sciences Co., Ltd., Ningbo, China). GCP was dissolved in distilled water and stirred overnight at room temperature to a solution of 1 mg/ml, then subjected to ultrasound treatment at a frequency of 20 kHz and 1,000 W power level in a certain pulse mode (5 s on and 5 s off) with different time. During the degradation process, a 6 mm diameter tip probe was immersed into the solution at 3 cm depth. Although Hu et al. (17) verified that the thermal effects during ultrasonic irradiation would not affect polysaccharide change if the internal temperature of ultrasonication was not over 100°C, the beaker of the polysaccharide solution was inserted into an ice-cold bath for avoiding the high temperature. When the ultrasonic time was sustained for 30 min, one group of the resulting solution was taken out and the other one was extended for 120 min degradation. After which, two ultrasonic degradation solutions were dialyzed against distilled water for 48 h (changed the water every 4 h and molecular weight cutoff was 8,000 Da). After that, GCP solutions were filtered through a 0.22 μm filter, applied to a Sepharose CL-6B column (2.5 cm × 60 cm) for further purification, and eluted with 0.1 mol/L NaCl solution at a flow rate of 0.6 ml/min. Then, the purified GCP fractions were respectively dialyzed against distilled water with a 3,500 Da dialysis bag for desalination. In the end, the dialyzed fractions were collected and freeze dried, the obtained polysaccharide fragments were named GCP-F1 (30 min) and GCP-F2 (120 min), respectively.

### Physicochemical properties analysis

#### Determination of monosaccharide composition

Genistein Combined Polysaccharide, GCP-F1, and GCP-F2 were dissolved in 2 mol/L TFA and hydrolyzed at 120°C for 2 h, respectively. The hydrolysate was washed three times with methanol and evaporated. Finally, the hydrolyzed material was transferred to a 25 ml volumetric flask, diluted to 25 ml with deionized water, and analyzed using high-performance anion exchange chromatography (HPAEC) according to the methods reported previously (1).

#### Determination of molecular weight

Genistein Combined Polysaccharide, GCP-F1, and GCP-F2 were dissolved to a concentration of 2 mg/ml, respectively. After which, their molecular weights were detected by high-performance size exclusion chromatography (HPSEC) according to the methods reported previously (1).

#### Fourier transform infrared spectroscopic analysis

Fourier transform infrared spectra of GCP, GCP-F1, and GCP-F2 were obtained using a Nexus 470 FT-IR Spectrometer (Nicolet, United States). The FT-IR spectra of GCP, GCP-F1, and GCP-F2 were recorded using a KBr pallet containing 0.1% GCP (GCP-F1 or GCP-F2), the wave number was set from 4,000 to 400 cm^–1^.

#### Nuclear magnetic resonance analysis

Genistein Combined Polysaccharide, GCP-F1, and GCP-F2 were dissolved in D_2_O in 5 mm NMR tubes, respectively, a Bruker Avance 500 MHz Spectrometer (Bruker Inc., Germany) was used to record the ^1^H NMR and ^13^C NMR spectra of GCP, GCP-F1, and GCP-F2 at 30°C. Chemical shifts for ^1^H NMR and ^13^C NMR spectra were recorded in parts per million.

### Antioxidant activity assay

#### 2,2′-azinobis-(3-ethylbenzothiazoline-6-sulfonic acid (ABTS) radical scavenging activity

2,2′-azinobis-(3-ethylbenzothiazoline-6-sulfonic acid radi-cal scavenging activities of GCP, GCP-F1, and GCP-F2 were detected according to the method reported previously (18). An equal volume of 7 mmol/L ABTS and 1.4 mmol/L potassium persulfate was mixed and stored in dark for 16 h at room temperature to prepare ABTS radical. Before use, ABTS radical solution was diluted with distilled water to an absorbance of 0.70 ± 0.02 at 734 nm. Then, 0.1 ml of polysaccharide solution with various concentrations (0.5, 1.0, 1.5, 2.0, 2.5, and 3.0 mg/ml) was added into 0.9 ml ABTS radical solution and mixed vigorously. After the mixture was reacted at room temperature for 5 min, the absorbance of the mixture was measured at 734 nm. ABTS radical scavenging activity was (%) = [1 – (A_*i*_ − A_*j*_)/A_0_] × 100%. Where A_0_ is the absorbance of control group without polysaccharide solution, A_*i*_ is the absorbance of polysaccharide solution, and A_*j*_ is the absorbance of background without ABTS radical. Meanwhile, Vc was used as a positive control in the following antioxidant tests.

#### 2,2-diphenyl-1-picrylhydrazyl radical scavenging activity

2,2-diphenyl-1-picrylhydrazyl radical scavenging activities of GCP, GCP-F1, and GCP-F2 were determined according to the method reported previously (14). DPPH was dissolved in alcohol to a concentration of 0.1 mmol/L, then 2 ml of polysaccharide solution with various concentrations (0.5, 1.0, 1.5, 2.0, 2.5, and 3.0 mg/ml) was added to 2 ml of alcoholic DPPH. The system was fully mixed and kept in dark for 30 min at room temperature. Finally, the absorbance of the mixture was measured at 517 nm with a microplate reader (Thermo, United States). DPPH radical scavenging activity was (%) = (A_0_ − A_*i*_ + A_*j*_)/A_0_ × 100%. Where A_0_ is the absorbance of the control group without polysaccharide solution, A_*i*_ is the absorbance of polysaccharide solution, and A_*j*_ is the absorbance of background without DPPH radical.

#### Superoxide anions scavenging activity

Superoxide anions’ scavenging activities of GCP, GCP-F1, and GCP-F2 were detected by pyrogallic acid method (2). Tris-HCl buffer of 2.5 ml (0.05 mol/L, pH = 8.2) was added into 0.4 ml of polysaccharide solutions with various concentrations (0.5, 1.0, 1.5, 2.0, 2.5, and 3.0 mg/ml). The mixture solution was reacted at 25°C for 10 min, then, 0.1 ml of pyrogallic acid was added and reacted for 20 min. The reaction was quenched by adding 0.5 ml of HCl and the absorbance of the mixture was measured at 380 nm. Superoxide anions scavenging activity was (%) = (1 − A/A_0_) × 100%. Where A is the absorbance of polysaccharide solution and A_0_ is the absorbance of the control group without polysaccharide solution.

#### Hydroxyl radical scavenging activity

Hydroxyl radical scavenging activities of GCP, GCP-F1, and GCP-F2 were detected according to the method reported by Hu et al. (19). In total, 2 ml of 6 mmol/L FeSO_4_ and 2 ml of 6 mmol/L H_2_O_2_ were added to 2 ml of polysaccharide solutions at various concentrations (0.5, 1.0, 1.5, 2.0, 2.5, and 3.0 mg/ml), the system was fully mixed and reacted at room temperature for 10 min. Then, 2 ml of 6 mmol/L ortho-hydroxybenzoic acid was added and reacted for another 30 min. The absorbance of the mixture was measured at 510 nm. Hydroxyl radical scavenging activity was (%) = (A_0_ – A_*i*_ + A_*j*_)/A_0_ × 100%. Where A_0_ is the absorbance of polysaccharide solution replaced by distilled water, A_*i*_ is the absorbance of polysaccharide solution, and A_*j*_ is the absorbance of H_2_O_2_ replaced by distilled water.

#### Antibacterial activity assay

Antibacterial activities of GCP, GCP-F1, and GCP-F2 against *Escherichia coli* and *Staphylococcus aureus* were assayed by the agar diffusion method (1). Polysaccharides were dissolved in distilled water to various concentrations (0.4, 0.8, 1.2, 1.6, and 2.0 mg/ml) and filtrated by 0.22 μm millipore filter. In total, 15 ml of nutrient agar was added to each glass plate and solidified, then 100 μl of 10^6^ CFU/ml of test organisms suspension was spread on the agar plate surface and a filter paper disk (diameter: 6 mm, thickness: 1 mm) containing 30 μl of polysaccharide solution was placed in the center of the plate. These glass plates were incubated at 37°C for 24 h. The antibacterial activities of GCP, GCP-F1, and GCP-F2 were evaluated by determining the diameters of inhibition zones with a vernier caliper.

### Statistical analysis

Data were expressed as means ± SD after triplicate repeats. Data were subjected to one-way ANOVA, and significant differences were analyzed using SPSS version 19.0 (IBM, United States).

## Results and discussion

### Ultrasonic degradation

**FIGURE 1:**
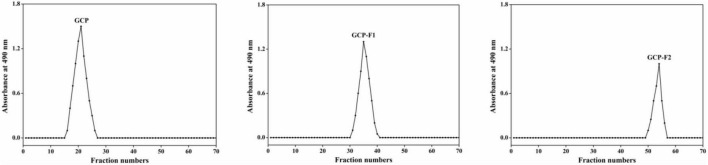
Elution curve of Genistein Combined Polysaccharide (GCP), GCP-F1, and GCP-F2 on Sepharose CL-6B column (2.5 cm × 60 cm).

As shown in Figure 1, after GCP is degraded by ultrasonic treatment at 20 kHz and 1,000 W power levels at different times, two elution curves (GCP-F1 and GCP-F2) are found on the separation and purification results of Sepharose CL-6B column, indicating that two polysaccharide fragments are obtained *via* ultrasonic treatment. Except for the regulations of metabolic pathway and fermentation conditions (13, 15), with enzymatic degradation, chemical degradation, physical degradation, and other degradation methods (20, 21), many researchers obtained similar de-polymerized polysaccharides to present work. Meanwhile, Yan et al. (22) demonstrated that polysaccharide fragments with different degrees of polymerization could also be obtained by controlling the treatment process and extraction methods.

### Effects of ultrasonic degradation on physicochemical properties

#### Monosaccharide composition

The composition of monosaccharides could affect the charge of polysaccharides, thus influencing their biological activities and physicochemical properties (23). Table 1 shows that the obtained ultrasonic fragments of GCP-F1 and GCP-F2 contain the same kind of neutral monosaccharides (arabinose, galactose, glucose, xylose, and mannose) and acid monosaccharide (glucuronic acid) as compared to GCP in the similar molar ratios. Interestingly, Yan et al. (24) found that monosaccharide composition of polysaccharides extracted from *Phellinus linteus* mycelia was not affected by ultrasonic treatment, and the molar ratios were slightly different. Wang et al. (25) demonstrated that monosaccharide composition and a molar ratio of yellow tea polysaccharide were almost not altered by ultrasonic degradation. Meanwhile, Yang et al. (26) also suggested that ultrasound treatment did not affect the monosaccharide composition and molar ratio of polysaccharides.

**TABLE 1:** Monosaccharide compositions and molecular weights of Genistein Combined Polysaccharide (GCP), GCP-F1, and GCP-F2.

Items	GCP	GCP-F1	GCP-F2
**Monosaccharide composition**			
Arabinose (μmol/L)	0.64	0.66	0.67
Galactose (μmol/L)	2.58	2.62	2.53
Glucose (μmol/L)	23.53	21.88	22.07
Xylose (μmol/L)	0.90	0.85	1.01
Mannose (μmol/L)	2.47	2.53	2.58
Glucuronic acid (μmol/L)	0.27	0.21	0.23
**Molecular weight**			
Weight-average molecular weight (Mw)	8.093 × 10^4^ Da	3.158 × 10^4^ Da	1.027 × 10^4^ Da
Number-average molecular weight (Mn)	7.982 × 10^4^ Da	3.117 × 10^4^ Da	1.016 × 10^4^ Da
Polydispersity (Mw/Mn)	1.014	1.013	1.011

#### Molecular weight

In general, polysaccharides with high molecular weight can form a gel layer on the surface of bacteria and exert better antibacterial activity (27), but low molecular weight polysaccharides usually have higher biological activities (28). As shown in Table 1, the weight-average molecular weight (Mw) of GCP is 8.093 **×** 10^4^ Da, Mw of GCP-F1 is decreased to 3.158 **×** 10^4^ Da with 30 min ultrasound treatment, and Mw of GCP-F2 is reduced to 1.027 **×** 10^4^ Da with 120 min ultrasonic time. Meanwhile, polydispersities of GCP, GCP-F1, and GCP-F2 were 1.014, 1.013, and 1.011, respectively. Many scholars found that the decrease in molecular weight could increase the physicochemical property and bioactivity of polysaccharides, such as rheological profiles, antitumor activity, antioxidant activity, moisture-preserving activity, and antibacterial activity (29, 30). However, Yang et al. (31) found that the anticancer activity of polysaccharides extracted from *Flammulina velutipes* was decreased with the reduction of their molecular weights. Meanwhile, Guo et al. (32) demonstrated that when the molecular weight of fucoidan was reduced too small by ultrasonic treatment, the antioxidant activity of which was also decreased.

#### Fourier transform infrared

**FIGURE 2:**
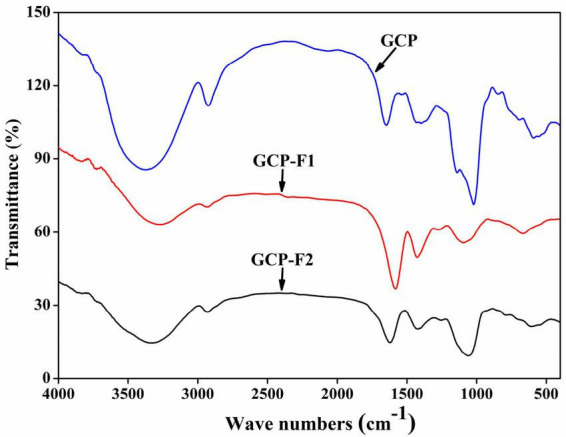
Fourier transform infrared (FT-IR) spectra of Genistein Combined Polysaccharide (GCP), GCP-F1, and GCP-F2.

As can be seen from Figure 2, the absorption peak between 3,400 and 3,200 cm−^1^ might attribute to O-H stretching vibration of hydrogen bonds (1), the absorption peak at around 2,940 cm − ^1^ might assign to asymmetric stretching vibration of C-H in CH, CH_2_, and CH_3_ groups (33), absorbance peaks at around 1,600 and 1,400 cm^–1^ might due to symmetrical C = O stretching vibrations and asymmetrical C = O stretching vibrations (2), absorbance peak at around 1,100 cm^–1^ might correspond to vibration absorption of C-O-H and C-O-C (34), and absorption peaks between 900 and 600 cm^–1^ might belong to β-glycosidic bonds and α-glycosidic linkages, respectively. However, Figure 2 shows that the intensity of the characteristic peaks in GCP, GCP-F1, and GCP-F2 was different, suggesting that the number and type of functional groups in GCP, GCP-F1, and GCP-F2 might be different and which will be analyzed in our future work.

#### Nuclear magnetic resonance

**FIGURE 3:**
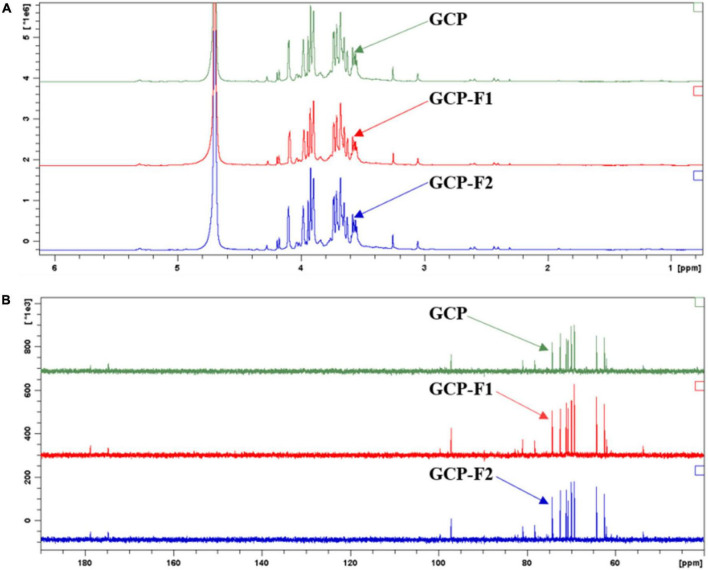
Nuclear magnetic resonance (NMR) spectroscopic analysis of Genistein Combined Polysaccharide (GCP), GCP-F1, and GCP-F2. **(A)** ^1^H-NMR; **(B)** ^13^ C-NMR.

As shown in Figure 3A of ^1^H-NMR chemical shift data, peaks between 3.5 and 3.6 ppm might belong to the CH2 linked to -OH in glucose and galactose, peaks between 3.6 and 3.8 ppm might relate to CH2 in the -O-CH2-OH of arabinose and the CH associated with -OH in monosaccharide, peaks between 3.8 and 4.0 ppm might attribute to CH in the -O-CH-COOH of glucuronic acid and in the HO-CH-CHO of glucose, and peak at about 4.1 ppm might relate to CH in the -O-CH-OH of monosaccharide, which was due to the movement of - O-, -OH, and -CHO-COOH as an electron-withdrawing group to the lower field with the connected CH. Meanwhile, hydrogen ions in -OH and -COOH of monosaccharides will be deuterated or dissociative in solvent D_2_O, thus no characteristic absorption peaks were shown on ^1^H NMR spectra (14). As can be seen from Figure 3B of ^13^ C-NMR results, peaks between 60 and 65 ppm might relate to the CH2 in glucose, arabinose, xylose, and galactose, peaks between 69 and 74 ppm might be due to the CH in monosaccharide, peak at 78.2 ppm might attribute to CH in the -O-CH-CH2OH of galactose, peak at 80.9 ppm might relate to CH in the -O-CH-COOH of glucuronic acid, peak at 97.1 ppm might relate to CH in the -O-CH-OH of monosaccharide, and peaks at 174.7 and 178.7 ppm might relate to CHO in glucose and COOH in glucuronic acid, respectively (1). The NMR spectra results of GCP, GCP-F1, and GCP-F2 indicated that they might have similar chemical structures.

### Effects of ultrasonic degradation on antioxidant activity

**FIGURE 4:**
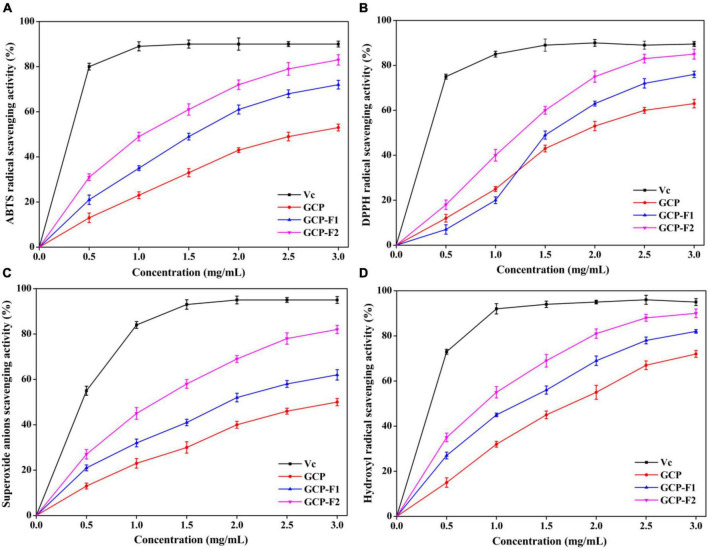
Scavenging effects of GCP, GCP-F1, and GCP-F2 on 2,2′-azinobis-(3-ethylbenzothiazoline-6-sulfonic acid (ABTS) radical **(A)**, 2,2-diphenyl-1-picrylhydrazyl (DPPH) radical **(B)**, superoxide anions **(C)**, and hydroxyl radical **(D)**.

As shown in Figure 4, GCP-F1 and GCP-F2 have higher antioxidant activities on ABTS radical, DPPH radical, superoxide anions, and hydroxyl radical in a concentration-dependent manner than those of GCP, though are lower than those of Vc. The scavenging activity order was GCP < GCP-F1 < GCP-F2 < Vc. At the concentration of 3.0 mg/ml, the maximum scavenging rates on ABTS radical, DPPH radical, superoxide anions, and hydroxyl radical were 53.18 ± 1.42%, 62.85 ± 1.91%, 50.04 ± 1.61%, and 71.93 ± 1.53% for GCP, 72.31 ± 1.88%, 76.04 ± 1.37%, 61.97 ± 2.35%, and 81.07 ± 0.86% for GCP-F1, and 83.55 ± 2.31%, 85.08 ± 2.21%, 82.16 ± 1.84%, and 90.08 ± 1.94% for GCP-F2, respectively. There might be many reasons for the increased scavenging activities of GCP-F1 and GCP-F2. On the one hand, ultrasonic degradation might not only increase hydroxyl groups and create new functional groups in GCP-F1 and GCP-F2 but also form radicals during water sonolysis, which will endow GCP-F1 and GCP-F2 had better antioxidant activities (21, 35). On the other hand, the content and ratio of arabinose, galactose, glucose, mannose, and glucuronic acid might also influence the antioxidant activity of GCP-F1 and GCP-F2 (36–38). Last but not least, GCP-F1 and GCP-F2 might have less compacted structures with the reduction of molecular weight after ultrasound treatment, which donated more electrons to react with free radicals (39, 40). However, the detailed action mechanism will be further analyzed in our future work.

### Effects of ultrasonic degradation on antibacterial activity

**FIGURE 5:**
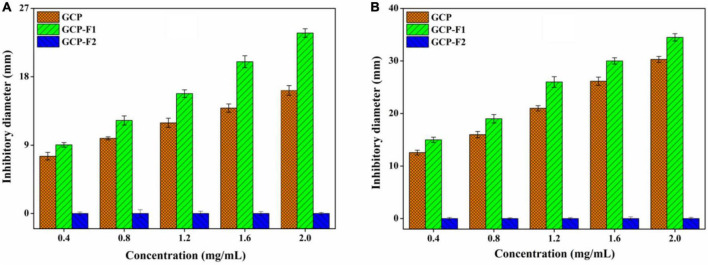
Antibacterial activities of Genistein Combined Polysaccharide (GCP), GCP-F1, and GCP-F2 against *E. coli* **(A)** and *S. aureus* **(B)**.

As can be seen from Figure 5A, GCP and GCP-F1 show a concentration-dependent manner against *E. coli*, and the inhibitory effect of GCP-F1 is higher than that of GCP. At the concentration of 2.0 mg/ml, inhibitory zones of GCP and GCP-F1 against *E. coli* were 16.23 ± 0.59 and 23.76 ± 0.51 mm, respectively, indicating that ultrasound treatment increased the antibacterial activity of GCP-F1. However, GCP-F2 almost lost its antibacterial activity against *E. coli* (Figure 5A). Meanwhile, Figure 5B shows a similar antibacterial trend of GCP, GCP-F1, and GCP-F2 against *S. aureus* to those of *E. coli*, inhibitory zones of GCP and GCP-F1 against *S. aureus* are 30.25 ± 0.48 and 34.51 ± 0.66 mm at 2.0 mg/ml but not for GCP-F2. Researchers found that hydroxyl and carboxyl groups in polysaccharide can chelate metal ions and influence the nutrient uptake of bacteria, thus playing its antibacterial activity (41). The increased antibacterial activity of GCP-F1 might be related to the more exposure to hydroxyl and carboxyl groups induced by the decrease of molecular weight and breaking of intermolecular hydrogen bonding (21, 42). Meanwhile, monosaccharide composition and the molar ratio will also affect the antibacterial activity of GCP-F1 and GCP-F2 (43). Furthermore, a low molecular weight of GCP-F1 might allow it to enter the bacteria and exert its antibacterial activity by affecting protein, nucleic acid, and energy metabolism of the cell (44–46). However, a too low molecular weight of GCP-F2 with further degradation prevents it from forming an active structure (13, 32), which might be the reason that GCP-F2 lost its antibacterial activity.

## Conclusion

Physicochemical property and biological activity of polysaccharide GCP and its fragments (GCP-F1 and GCP-F2) obtained by ultrasonic degradation were analyzed. Compared to GCP, a lower molecular weight of GCP-F1 and GCP-F2 showed higher antioxidant activities in a concentration-dependent manner. Though ultrasonic degradation increased the antibacterial activity of GCP-F1 by reducing its molecular weight, GCP-F2 almost lost its antibacterial activity with further ultrasonic degradation and molecular weight reduction. Therefore, there might also be specific active units in polysaccharides as compared to an enzymatic process, and it is very important to obtain appropriate polymerization of polysaccharides for their better bioactivities.

## Data availability statement

The original contributions presented in this study are included in the article/supplementary material, further inquiries can be directed to the corresponding author/s.

## Author contributions

SL contributed to conception, design, and funding of the study. YW, WD, and WH organized the database. CX wrote the first draft of the manuscript. QS and ZW contributed to writing—review and editing. All authors approved it for publication.

## Conflict of interest

The authors declare that the research was conducted in the absence of any commercial or financial relationships that could be construed as a potential conflict of interest.

## Publisher’s note

All claims expressed in this article are solely those of the authors and do not necessarily represent those of their affiliated organizations, or those of the publisher, the editors and the reviewers. Any product that may be evaluated in this article, or claim that may be made by its manufacturer, is not guaranteed or endorsed by the publisher.
